# Graphene aerogels: part 1 - derived from graphene oxide and thermally reduced graphene oxide via supercritical carbon dioxide drying

**DOI:** 10.55730/1300-0527.3657

**Published:** 2024-02-08

**Authors:** Meryem SAMANCI, Ayşe BAYRAKÇEKEN

**Affiliations:** 1 Department of Chemical Engineering, Faculty of Engineering, Atatürk University, Erzurum, Turkiye; 2 Department of Nanoscience and Nanoengineering, Atatürk University, Erzurum, Turkiye

**Keywords:** Graphene oxide, thermally reduced graphene oxide, graphene aerogel, sol-gel method, carbon corrosion, pseudo-capacitive charge

## Abstract

Graphene aerogels have become promising materials in many areas of industry, especially in energy applications due to their superior physical and electrochemical properties. Generally, graphene oxide (GO)-derived aerogels (A) are synthesized by using the hydrothermal method. In this study, both GO and reduced graphene oxide (RGO)-derived aerogels were synthesized by using the sol-gel method coupled with the supercritical carbon dioxide (SCCO_2_) drying process. It aims to examine the changes in the structure of the final aerogel by changing the amount (0.25–0.5–1% wt.) and type of graphene-based precursor materials used in the synthesis. Physical characterizations of graphene aerogels were conducted using Brunauer-Emmett-Teller (BET) analysis, scanning electron microscope-energy dispersive X-ray (SEM-EDX) analysis, transmission electron microscopy (TEM), micro-Raman spectroscopy, X-ray diffractometer (XRD) to highlight their structural properties. Additionally, X-ray photoelectron spectroscopy (XPS) analyses were performed to determine the oxidation levels on the surface of the RGO-1 aerogel. The cyclic voltammetry (CV) method was used to examine the electrochemical behavior of the graphene aerogels against corrosion. Specific capacitance values of the synthesized materials were calculated before and after corrosion. Furthermore, the surface charge changes that occur after corrosion were examined. GOAs displayed the highest specific capacitance value among graphene aerogels. Notably, the RGOA-1 aerogel exhibited the highest corrosion resistance. The pseudo-capacitive charge ratio of RGOA-1 after corrosion was measured at 0.5 mC cm^−2^.

## 1. Introduction

Carbonaceous aerogels are promising materials for electrodes and catalyst supports due to their characteristics such as high porosity, high surface area, low density, and 3D network structure. Due to the unique surface properties and porous structure of carbonaceous aerogels, they contain many active sites. Therefore, diffusion/mass transfer of liquid or gas phases and adsorption mechanisms of ions or molecules occur with high efficiency [1]. Carbonaceous aerogels are generally classified into four groups: polymer-derived aerogels [2], carbon nanotube-based aerogels [3], graphene-based aerogels [4], and biomass-derived aerogels [5]. On the other hand, hybrid aerogels synthesized from any two-carbonaceous aerogel have also been investigated [6–8].

Graphene aerogels are 3D network structures formed by interconnected graphene sheets. They are synthesized using 2D graphene and graphene-based precursor materials that have undergone surface modification, resulting in extraordinary physicochemical properties [9]. Graphene aerogels with high porosity and low density are formed through the stacking of graphene sheets that self-arrange and cluster due to π-π interactions between them [10]. Graphene aerogels are used in numerous applications due to their fascinating properties such as high mechanical strength, electrical conductivity, thermal resistance, adsorption capacity, and good compressibility/expandability [11]. Because of these properties, it is applied in areas such as energy storage systems [12,13], fuel cells [14], catalysts [15], photocatalysts [16], sensors [17], environmental protection [18], biomedical [19].

In general, graphene aerogels are synthesized in five different ways: hydrothermal reduction/self-assembly [20], chemical reduction [21], template-directed reduction [22], crosslinking [23], and sol-gel [24] processes. Additionally, graphene aerogels can be synthesized using sol-gel chemistry by forming physical crosslinks between graphene sheets. When synthesizing graphene aerogels, various drying methods can be used after the sol-gel process: drying with supercritical fluid (aerogel), freeze-drying (cryogel), and drying under ambient conditions (xerogel). These differences in the synthesis stages of graphene aerogels enable us to synthesize solid nanomaterials in which the pore distributions in the structure can be easily adjusted. Lim et al. synthesized graphene aerogel using the sol-gel method. They developed an ultrafast method based on the polycondensation of resorcinol, formaldehyde, and graphene oxide using hydrochloric acid (HCl) as the catalyst and acetonitrile (AN) as the solvent. After supercritical CO_2_ drying and pyrolysis processes, the electrochemical capacities of the synthesized graphene aerogels and their capability to absorb toxins (cyclohexane) were investigated [24]. Liu et al. obtained an organic gel through the polycondensation of phenol-formaldehyde and the utilization of GO as the crosslinking agent. The wet gel was converted into graphene xerogel by drying under ambient pressure [25]. Xia et al. prepared porous graphene cryogels with high specific surface areas by freeze-drying and the assembly of GO and resorcinol-formaldehyde monomers. The capacitive properties of the prepared graphene cryogels were investigated [26]. Nagy et al. synthesized polymer hydrogels based on resorcinol-formaldehyde with varing levels of GO content, using sodium carbonate (Na_2_CO_3_) as a catalyst. Hydrogels were converted into graphene aerogels after supercritical CO_2_ drying and heat treatment in a nitrogen (N_2_) atmosphere. They observed that the amount of GO in the synthesized materials did not affect the morphology of the polymer and carbon form of the materials, while their specific conductivity increased linearly [27].

GO is used as the starting material in the synthesis of graphene aerogel [28]. Due to its hydrophilic structure, GO is highly dispersed in aqueous media. Moreover, the structural functionality of GO, which includes oxygen-containing functional groups (hydroxyl, epoxy, carbonyl, and carboxyl groups) in the basal plane and at its edges, makes it suitable for synthesizing 3D network-shaped structures [29].

In some studies, reduced graphene oxide (RGO) is used instead of GO in graphene aerogel synthesis. Chemical or thermal reduction techniques are generally used in RGO synthesis [30]. Alam et al. synthesized GO using the Hummer method and RGO through the thermal reduction method. They reported that GO layers are thicker than RGO due to the functional groups present on their edges. As a result, the average particle size distribution is higher. They also observed that GO was more hydrophilic than RGO due to these structural changes [31]. These RGOs used in graphene aerogel synthesis are generally carried out by using chemically reduced graphene oxide in solution [4,32]. The chemical reduction technique, which employs the use of a liquid medium, can pose challenges for the industrial scale production of RGO. The thermal reduction technique is suitable for industrial production, which has a high reduction degree, is environmentally friendly, inexpensive, and can produce large quantities [30]. In this study, different from the literature, graphene aerogels with different surface properties were synthesized by using both GO and thermally reduced graphene oxide (RGO) in different ratios as graphene precursor material. The effects of GO and RGO, which have functional groups containing oxygen in different ratios (degree of hydrophilicity or hydrophobicity), on the structures of graphene aerogels were investigated.

Graphene aerogels are ideal materials for energy systems due to their fast electron/ion transport, superior chemical and physical stability against ambient conditions, and porous hierarchical structure that provides good cycling performance [10]. However, graphene aerogels, which are a type of carbonaceous aerogels, may encounter the issue of carbon corrosion. Insufficient resistance of the electrode material to corrosion leads to high costs, especially negatively affecting the commercialization of fuel cells. To address this issue, numerous studies have been conducted in the literature. Most commonly, the degree of graphitization of the material is increased to improve tolerance to carbon corrosion [33]. Wang et al. have synthesized carbon nanolattices (CNCs) as a supporting material for electrodes in hydrogen oxidation reaction (HOR) and oxygen reduction reaction (ORR). During the synthesis process, the level of graphitization was altered by varying the pyrolysis temperature, and in situ N doping was achieved by using an N-containing carbon precursor. Platinum catalysts are placed on the surface of the support material. The electrocatalyst, supported by CNCs with high graphitization degree and N doping, exhibited high catalytic activity and stability due to its strong oxidation resistance [34]. Avasarala et al. investigated the electrochemical oxidation of carbon black by simulating the operating conditions of proton exchange membrane fuel cells. As a result of the oxidation process, they observed that quinone and hydroquinone (Q-HQ) functional groups were formed on the carbon black surface, and there was an increase in the total surface oxygen [35].

Various approaches have been proposed in the literature for synthesizing graphene aerogels, resulting in different structural properties. This diversity in pioneering materials adds originality to this work. In this study, graphene aerogels were synthesized using the sol-gel method and subsequently subjected to supercritical drying. The physical properties of the synthesized materials were analyzed using various techniques including Brunauer-Emmett-Teller (BET) analysis, scanning electron microscope-energy dispersive X-ray (SEM-EDX) analysis, transmission electron microscopy (TEM), micro-Raman spectroscopy, X-ray diffractometer (XRD), and X-ray photoelectron spectroscopy (XPS). Additionally, cyclic voltammetry (CV) analyses were performed to examine the electrochemical behavior of the synthesized graphene aerogels against carbon corrosion.

## 2. Materials and methods

### 2.1. Experimental materials

During the GO synthesis process, graphite (flake, 325 mesh, Alfa Aesar, Ward Hill, MA, USA), phosphorus pentoxide (P_2_O_5_, ≥98%, Sigma-Aldrich, St. Louis, MO, USA), potassium persulfate (K_2_S_2_O_8_, ≥99.9%, Sigma-Aldrich), potassium permanganate (KMnO_4_, 99%, Merck, Darmstadt, Germany), sodium nitrate (NaNO_3_, ≥99.9%, Sigma-Aldrich), sulfuric acid (H_2_SO_4_, 95–98%, Merck), hydrogen peroxide (H_2_O_2_, 30%, Merck) and hydrochloric acid (HCl, 37%, ISOLAB, Eschau, Germany) were used. Argon (Ar) gas was used to synthesize thermal RGO. For aerogel synthesis, pure water, 99% resorcinol (C_6_H_4_(OH)_2_, Sigma-Aldrich), 34.5% formaldehyde (CH_2_O, Sigma-Aldrich), as catalysts 99% sodium carbonate (Na_2_CO_3_, Sigma-Aldrich) and acetone (99.5%, Sigma-Aldrich) were used. Carbon dioxide (CO_2_) was used as the supercritical fluid in the supercritical drying process. Nafion solution (15%, Ion Power, Inc., Tyrone, PA, USA) and 1,2-propanediol (C_3_H_8_O_2_, ≥ 99.5%, Sigma-Aldrich) were used for electrode solutions. Perchloric acid (HClO_4_, 70–72%, ISOLAB) was used as the electrolyte in electrochemical analysis. High-purity N_2_, Ar, and CO_2_ gases purchased from Habaş Company (Ankara, Türkiye) were used in various synthesis and analysis steps.

### 2.2. Synthesis of GO and RGO

The Hummer method was employed to synthesize GO, following the procedure outlined in our previous study [36]. The synthesis process involves two stages. In the first stage, preoxidation was carried out using commercial graphite and oxidizers such as P_2_O_5_, K_2_S_2_O_8_, and H_2_SO_4_, prior to proceeding to the Hummer method. Subsequently, in the second stage, a mixture of 3 g of graphite oxide, 69 mL of H_2_SO_4_, and 1.5 g of NaNO_3_ was prepared in a beaker. The mixture was placed in an ice bath, and 9 g of KMnO_4_ was slowly added to ensure that the temperature remained below 5 °C. The mixture was stirred in the ice bath for 30–45 min, followed by removal from the ice bath and stirring at 35 °C for an additional 3 h. During the ice bath, 138 mL of distilled water was added gradually to the mixture at a solution temperature of approximately 50 °C. After stirring for 30 min, 420 mL of distilled water were added. Subsequently, 7 mL of 30% H_2_O_2_ was added dropwise to the mixture. Washing steps were performed using 250 mL of 10% HCl solution followed by two rinses with distilled water. The resulting GO was then dried in an oven at 60 °C for 24 h.

RGO closely resembles the original graphene, and reduction is the most significant reaction of graphene oxide. The reduction of GO reduction can be achieved through thermal (annealing, microwave, light), chemical (using a reducing agent, photocatalyst, electrochemistry, hydrothermal reaction), or a combination of these methods [30]. In this study, RGO was synthesized through thermal reduction of GO at 500°C [36]. RGO has a structure that lies between GO and graphene, depending on the number of graphene layers and the ratio of functional groups present. Several studies have reported that as the pyrolysis temperature is increased, especially above 500 °C, the distance between graphene layers narrows considerably and the number of layers of graphene layers gradually decreases due to intense thermal exfoliation [37]. This is due to the thermal removal of oxygen-containing functional groups on the graphene layers in proportion to the increase in temperature [37].

The determined amount of GO was placed in the tubular furnace and kept in an Ar atmosphere at 500 °C for 2 h. The heating rate of the oven is set at 10 °C per minute. Ar gas was continuously supplied until the RGO cooled to room temperature.

### 2.3. Synthesis of graphene aerogel

Synthesis of graphene aerogels was carried out by sol-gel method. Aerogels are synthesized by crosslinking the functionalized surfaces of GO or RGO with hydroxymethyl groups formed by the polymerization of resorcinol (R), and formaldehyde (F) in the presence of a catalyst (C, Na_2_CO_3_). Typical sol-gel parameters such as ambient temperature, pH, and concentrations of reactants affect the final structure. The molar ratios of the reactants in the sol solution are as follows: R/C at 100, R/W at 0.02, and R/F at 0.5. Prior to the addition of these reactants, graphene-based precursor materials (GO or RGO) were mixed in a beaker for 30 min at ratios of 0.25% (GOA-1 or RGOA-1), 0.5% (GOA-2 or RGOA-2), and 1% (GOA-3 or RGOA-3) by mass of the total solution, considering the calculated masses of reactants (R, F, C, W) based on 20 mL of pure water (W). After thoroughly dissolving the GO and RGO precursors in pure water, resorcinol, formaldehyde, and catalyst were sequentially added to the solution. The initial pH of the solution was approximately 3.5. However, after adding the catalyst to the sol solution, the pH increased to approximately 7.8. These pH values vary depending on the amount of graphene-based precursor material added to the sol solution and the amount of oxygen-containing functional groups in the graphene-based material. These pH values are in the appropriate range that allows gelation to occur [38,39]. The solution was stirred for 6 h at approximately 35 °C, without exposure to the environment. The mixture was then placed in a sealed tube and kept in an oven at 50 °C for 24 h, followed by 72 h at 90 °C. The formation of the aerogel network in this gelation step involves two basic stages: hydrolysis of precursor materials and condensation of primary nanoparticles. During the first stage, hydroxyl groups are formed by the precursors. During the second stage, crosslinking reactions occur until a mechanically stable network structure is developed [40]. At the end of the fourth day, the wet gels were removed from the tube, transferred into acetone, and kept in acetone for 48 h for solvent exchange. Organic aerogels were then synthesized by extracting acetone from the pores of the materials using supercritical carbon dioxide (SCCO_2_) under a pressure of 17.24 MPa in a high-pressure reactor at 50 °C.

The final step in the preparation of graphene aerogels from organic aerogels is the pyrolysis process. In this process, the organic aerogel is pyrolyzed using a heat treatment to remove oxygen- and hydrogen-containing groups. During the pyrolysis process, it leads to the formation of a highly interconnected network structure [41]. Pyrolysis temperature has a direct effect on the final properties of aerogels. In this process, the pore volume of the final aerogel increases with increasing temperature. As the carbonization temperature increases, the porous structure of the gel is expected to change, resulting in more micropores. This is due to the release of more volatile matter at higher temperatures. In short, the pyrolysis process activates and increases the surface area of the synthesized materials [41]. Graphene aerogels were synthesized by the pyrolysis of organic aerogels, which were placed in a tubular furnace at 1000 °C in an N_2_ environment, with a heating rate of 15 °C min^−1^ for 4 h. Literature studies have reported that a pyrolysis temperature of 1000 °C is optimal for obtaining a high surface area. Wiener et al. found that the surface area and micropore volume of resorcinol-formaldehyde-derived aerogels increased up to 1000 °C, but decrease above this temperature [42].

**Figure 1:**
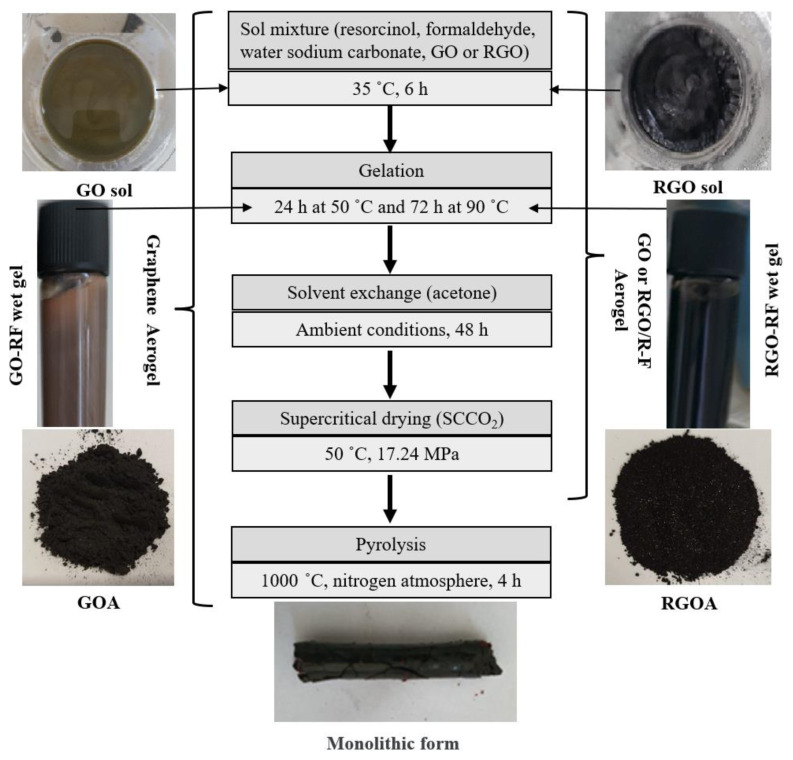
Synthesis steps of graphene aerogels (GOA-2 and RGOA-2).

Figure 1 summarizes the synthesis steps of graphene aerogels. The sol shows visible color changes of graphene-based precursor materials (GO or RGO). The powder structure of the final GOAs has a grainy consistency. In contrast, the powder structure of the final RGOAs is in the form of particles.

## 3. Characterization

### 3.1. Physical characterizations of materials

Physical characterization methods were used for the determination of several properties of the materials. The Brunauer-Emmett-Teller (BET) method and N_2_ adsorption/desorption isotherm analysis were used to obtain the surface area, porosity, and pore size distributions of the materials using a Micromeritics 3Flex 3-port BET surface area device. Scanning electron microscope-energy dispersive X-ray analysis (SEM-EDX, Zeiss Sigma 300, magnification ratio: 10×–1.000.000×) was used to obtain information about the topography and composition on the material surface. The microscopic morphologies of some materials have been characterized by transmission electron microscopy (TEM), specifically the Hitachi HighTech HT7700, with resolution values ranging from 0.204 nm (100 kV) to 0.144 nm (120 kV). Additionally, Raman spectroscopy (WITech alpha 300R, display type: 2D-3D, wavelength range: 350–1050 nm) was used to obtain information about the bonds formed by the atoms or molecules in the materials. Phases of materials, number of phases, crystal size, lattice parameters, and changes in structure were determined using X-ray diffractometry (XRD, PANalytical Empyrean) in the range of 10° ≤ 2θ ≤ 90°. Additionally, X-ray photoelectron spectroscopy (XPS, Specs-Flex XPS, energy range: 200 eV–4 keV) analyses were performed to determine the oxidation levels on the surface of the RGO-1 aerogel.

### 3.2. Electrochemical characterizations of the materials

Electrochemical characterization of graphene aerogels was carried out by cyclic voltammetry (CV) method. VersaSTAT 3 potentiostat device (AMETEK Scientific Instruments, Oak Ridge, TN, USA) was used for these characterizations. Additionally, it was combined with the rotating disc electrode system supplied by Pine Instrument (Grove City, PA, USA). CV analyses were performed with a standard three-electrode electrochemical cell system consisting of working (glassy carbon (GC), 0.1963 cm^2^), reference (Ag/AgCl), and counter (Pt wire) electrodes. Analyses were performed in 0.1 M perchloric acid (HClO_4_) electrolyte, which is the most commonly used electrolyte. This is because the anion of the electrolyte can block the active centers of the catalyst or change the binding energies of intermediate products in the reactions. Perchlorate anions (ClO_4_^−^) are adsorbed on the surface of metal catalysts at a minimum value compared to other electrolyte anions (HSO_4_^−^, SO_4_^2−^, H_2_PO_4_^−^, Br^−^) [43]. In addition, an electrolyte concentration of 0.1 M is generally preferred due to its intermediate value between minimum pollution and high conductivity [44].

In preparing the electrode inks of the materials undergoing corrosion testing, the material synthesized at a mass loading rate of 28 μg cm^−2^, 1 mL of pure water, 1 mL of 1,2-propanediol, and 165 μL of Nafion solution were used. Subsequently, this ink was homogenized using a homogenizer (ULTRA-TURRAX, IKA, Königswinter, Germany), and 5 μL of the resulting mixture was applied to the working electrode, then allowed to dry under ambient conditions.

N_2_ gas was injected into the electrolyte solution for 30 min to remove dissolved oxygen without taking CV measurements. During the corrosion process, N_2_ gas was put onto the surface of the electrolyte solution to ensure an inert test environment while measurements were taken. The material deposited on the working electrode underwent a 24-h corrosion treatment. Electrochemical oxidation processes were conducted by applying a constant potential of 1.2 V. CV analyses were performed at various scan rates (20, 50, 100 mV s^−1^) within the potential range of −0.28 to 0.92 V, and within different potential ranges (−0.28 to 0.4, 0.6, 0.8, 1 V) at a constant scan rate of 50 mV s^−1^. Measurements were taken both before and after corrosion. Current density values were calculated based on the geometric area of the electrode. Current-voltage curves were plotted based on the analysis results. Electrochemical impedance spectroscopy (EIS) analyzes the frequency range between 1 and 100 kHz at a constant potential value of 0.9 V. The Nyquist plots (imaginary impedance-real impedance) before and after corrosion were examined based on these data.

## 4. Results and discussion

BET, SEM-EDX, XRD, FTIR, RAMAN analyses of graphite, synthesized GO and RGO, digital photographs of materials and representatives of syntheses are provided in our previous study [36]. BET, SEM-EDX, XRD, and RAMAN analyses of GOAs and RGOAs were performed. The structural properties (such as surface area, pore diameters, and pore size distribution) of the materials were obtained with the Brunauer-Emmett-Teller (BET) method and N_2_ adsorption/desorption isotherm analysis. Table 1 presents the multipoint BET surface area, BJH pore volume, and pore size distribution values of the materials. In our previous study, GO was synthesized using commercial graphite powder by the Hummer method [36]. Subsequently, the RGO structure was obtained by thermal reduction to GO [36]. After the reduction, the BET surface area increased from 10.51 m^2^ g^−1^ to 213.37 m^2^ g^−1^. The surface structure of GO is hydrophilic due to the presence of oxygen-containing functional groups, whereas RGO has a more hydrophobic structure as it contains fewer functional groups [31,45]. These structural differences change the distribution and crosslinking mechanism of graphene in the starting mixture. This situation changes the structural properties of graphene aerogel considerably. Graphene aerogels were synthesized as a single structure with a large surface area by covalent bonds formed between the polymerized organic precursors and the oxygen-containing functional groups in GOs and RGOs and their simultaneous carbonization and thermal reduction processes [46]. For example, the specific surface areas of graphene aerogels vary due to differences in the amount of oxygen-containing functional groups in the reactant solution during the synthesis of RGOAs and GOAs. This leads to a change in the crosslinking mechanism of the gel. As the GO ratio in the aerogel mixture increased, the surface area decreased. On the other hand, as the ratio of RGO in the mixture increased, the surface area increased. The surface areas of graphene aerogels synthesized with RGO obtained by thermal reduction of GO increased considerably. Among GOAs, GOA-1 has the highest surface area, measuring 470.56 m^2^ g^−1^, while among RGOAs, RGOA-3 has the highest surface area, measuring 923.43 m^2^ g^−1^. Apart from that, due to the precursor GO present in the GOAs, the average pore size is smaller than that of the RGOAs, while the average particle size is larger due to more intense crosslinking occurring during gelation.

**Figure 2:**
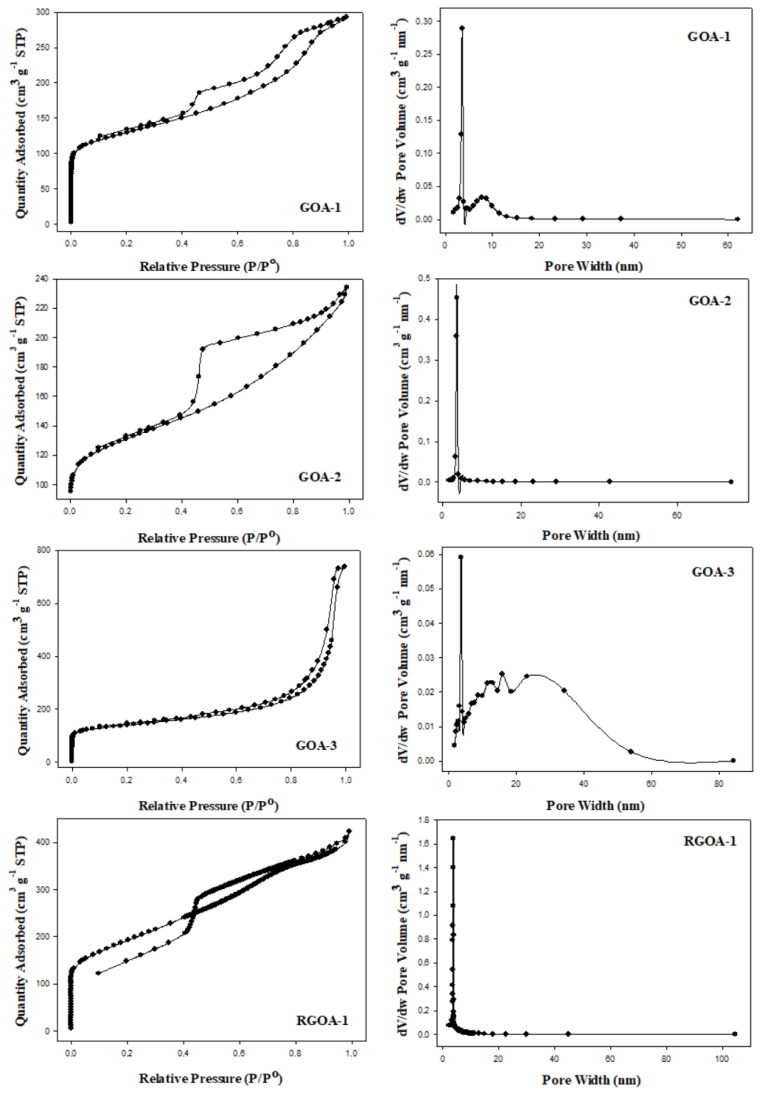

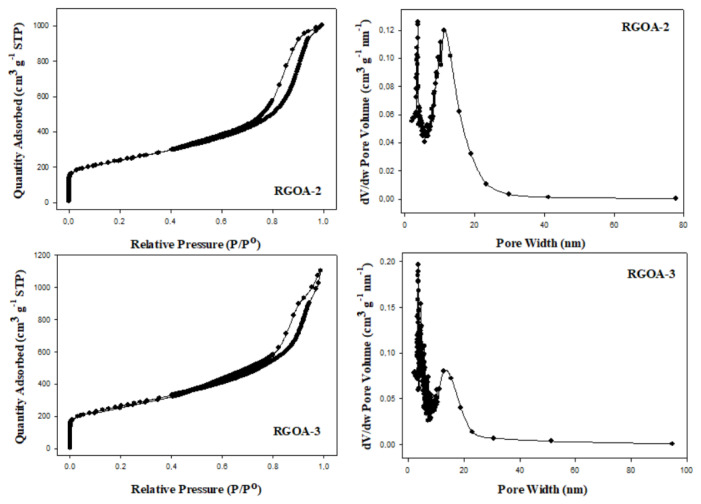
N_2_ adsorption-desorption isotherms and pore size distributions of graphene aerogels.

Graphene aerogels are 3D materials that incorporate the superior physical and chemical properties of both graphene and aerogels. These properties of the material determine the potential application areas of graphene aerogels, including their specific surface areas, porosity, and pore size distributions. The surface properties of graphene aerogels can vary significantly depending on various factors such as synthesis methods, reaction parameters (pH, ambient temperature, mixing speed, and time), type and ratio of reactants, structure and amount of graphene-based precursor material, drying method, and pyrolysis conditions. The micropores in the aerogel are related to the intraparticle structure, while the meso- and macropores are related to the interparticle structure. The size and shape of the precursor nanoparticles can be used to control the porosity of the material [47]. In some studies in the literature, graphene aerogel syntheses were carried out using the sol-gel method and GO as a graphene-based precursor material. The parameters of the synthesis steps and surface properties of some graphene aerogels reported in the literature are summarized in Table 2. Based on these studies, it is evident that GO has been the preferred graphene-based precursor material in the sol-gel method thus far. However, this study used RGO as a graphene-based precursor material, which differs from the literature. Using N_2_ adsorption/desorption isotherms and the Barrett-Joyner-Halenda (BJH) method, the pore volume and pore size of the materials were analyzed from the desorption branches of the isotherms, and the results are shown in Figure 2. It was observed that graphene aerogels conform to type IV isotherm and exhibit an H2 type hysteresis curve according to the classification made by IUPAC [55].

The type IV isotherm indicates that the structure is mostly composed of mesopores. The presence of H2-type hysteresis is also a characteristic of spherical agglomerated systems. However, solids exhibiting such hysteresis do not have a well-defined distribution of pore size and shape [56]. The size of hysteresis loops increased with the total adsorption of RGOAs compared to GOAs. This is because the BET surface areas and BJH pore volumes of RGOAs are higher than those of GOAs [48]. The mean pore diameter decreased as the amount of GO increased in GOAs and increased as the amount of RGO increased in RGOAs.

**Figure 3:**
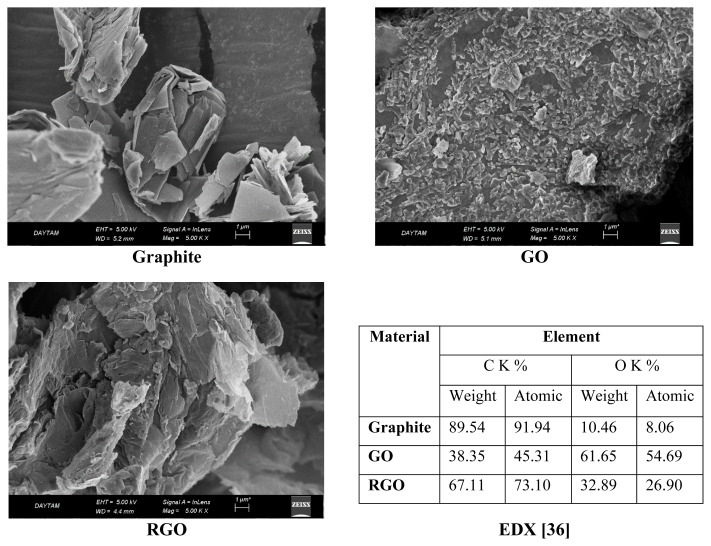
SEM images and EDX analyses of commercial graphite, GO and RGO.

SEM images and EDX analyses of commercial graphite, GO, and RGO are presented in Figure 3. In SEM images, it is seen that when GO is synthesized from graphite, which exists in the form of graphene stacks, the graphene plates become smaller layers and the number of layers decreases. In the RGO obtained after the thermal reduction of GO, it is observed that the graphene sheets exhibit greater expansion and the number of layers decreases considerably compared to commercial graphite. The EDX analysis revealed that the weight percentage of oxygen increased from 10.46% in graphite to 61.65% in GO and 32.89% in RGO, indicating successful exfoliation and thermal reduction process. The atomic oxygen ratio in RGO is approximately 50% less than in GO. Thus, through the thermal reduction process, the oxygen-containing functional groups, which are quite high in GO, are considerably reduced.

**Figure 4:**
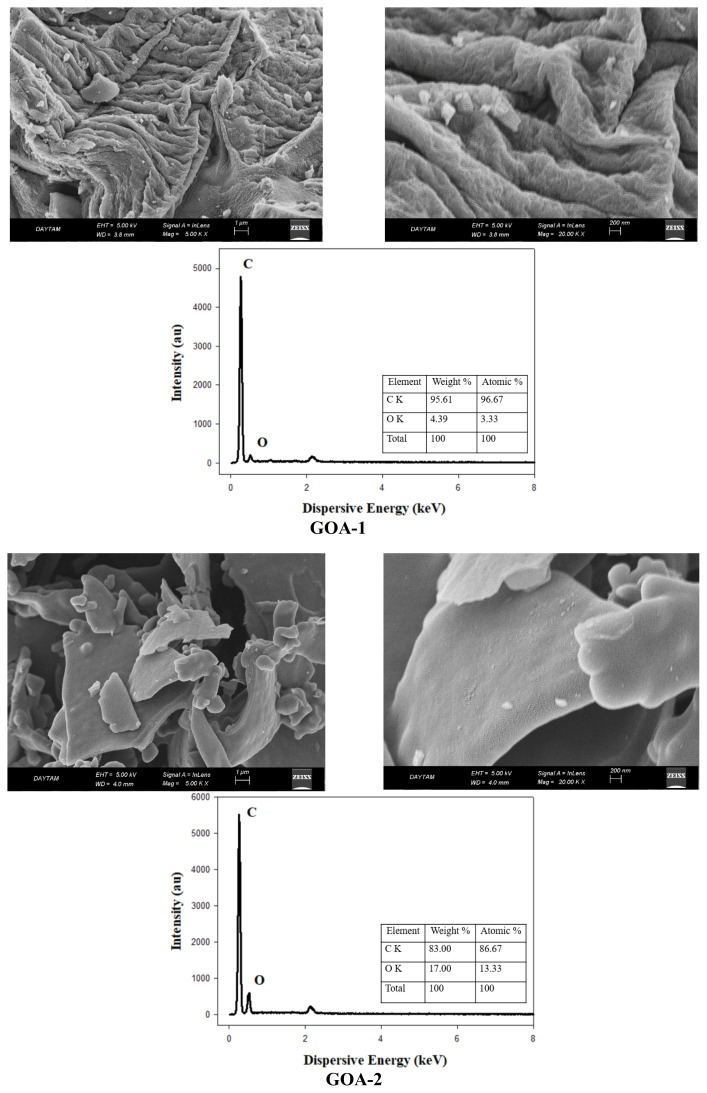

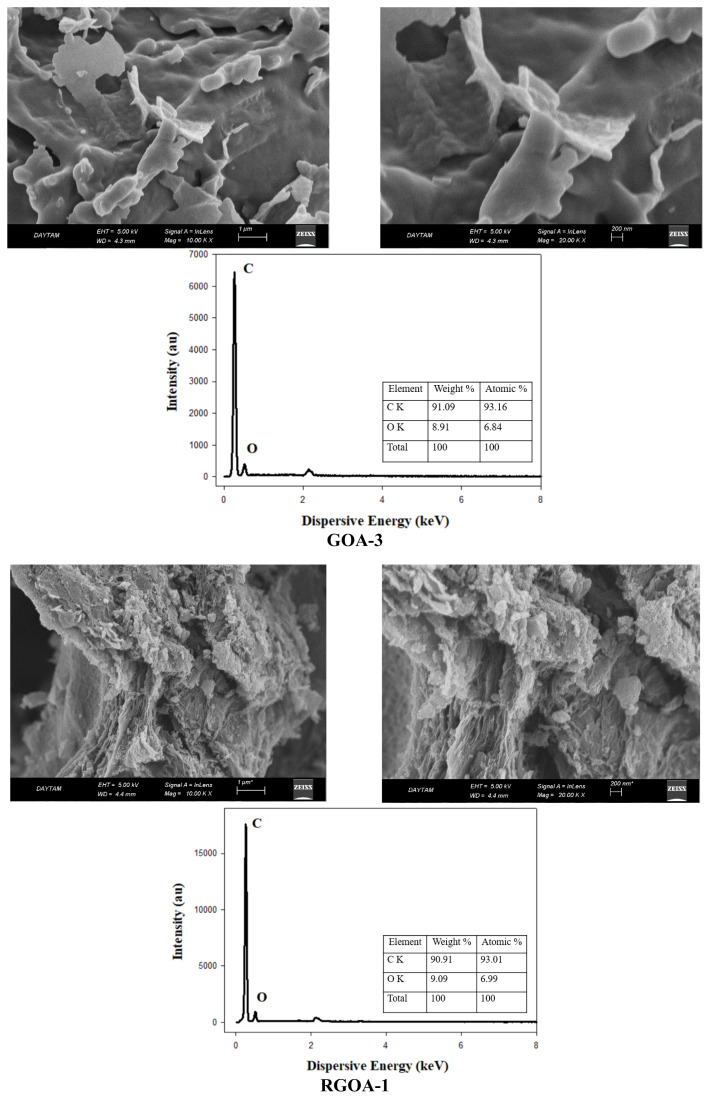

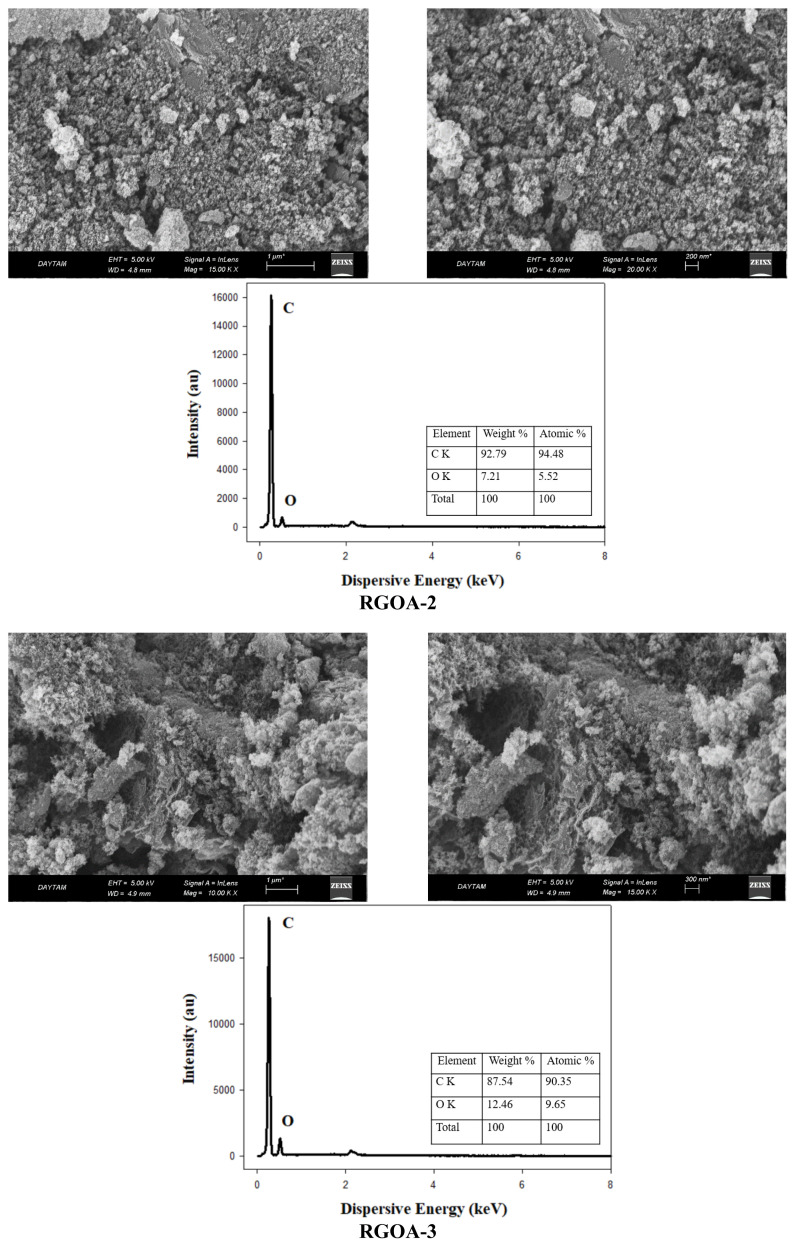
SEM images and EDX analyses of graphene aerogels.

SEM images of graphene aerogels are presented in Figure 4. It is seen that the surface morphologies and textural structures of graphene aerogels (GOAs and RGOAs) synthesized in two groups differ significantly from each other. When R-F is added to the solution of graphene-based precursors (GO and RGO), chemical and physical interactions occur between R-F molecules and graphene-based materials. The chemical interaction is due to chemical bonds formed by the bonding of oxygen atoms in hydroxyl groups on the surface of graphene-based materials and carbon atoms in formaldehyde in the presence of a catalyst. The physical interaction is due to the π–π stacking between the clustered R-F chains and the layers of graphene-based materials [49]. At the lowest GO ratio, the GO layers were completely embedded in the R-F molecules during synthesis. GOA-1 has a randomly oriented platelet structure in the form of crumpled papers. Plate planes increased as the GO ratio in the aerogel increased. As the amount of GO increased, the R-F molecules could not completely surround the surface of the GO sheets but instead grew along the sheet surfaces. GO plates and clustered R-F chains on these plates are clearly visible in the GOA-2 and GOA-3 aerogels [49]. In RGOAs, the aerogel structure with the lowest RGO ratio (RGOA-1) consists of spherical carbon particles dispersed between graphene sheets. As the ratio of RGO in the aerogel increases, the ratio of spherical carbon particles on the surface of the plate planes also increases. The surface area also increased proportionally with the increase of the RGO ratio. With the high spherical particle ratio in RGOA-3, the BET surface area reached up to 923.43 m^2^ g^−1^. The GO and RGO sheets served as the template for the 3D network-shaped structure of the graphene aerogel. It is seen that all graphene aerogels exhibit different 3D network structures and different pore properties from each other. Liu et al. observed that phenol-formaldehyde clusters form very thin interconnected nanoparticles on the GO surface in their phenol-formaldehyde-derived graphene xerogels [49]. Xia et al. synthesized the graphene cryogels and observed that GO layers could not provide sufficient polymerization zone in materials with RF/GO ratio up to 100:1. They reported that the R-F polymers in the mixture self-nucleate, carbon nanoparticles with a diameter of approximately 50 to 100 nm are dispersed on the surface of the graphene sheets [26]. EDX spectra of graphene aerogels are presented in Figure 4. The graphene aerogel with the highest carbon ratio is GOA-1, and the graphene aerogel with the highest oxygen ratio is GOA-2. In general, the carbon content in graphene aerogels is above 80%. The C/O ratios of precursor graphene-based materials and graphene aerogels are summarized in Table 3. The highest C/O ratio belongs to GOA-1 among GOAs, while it belongs to RGOA-2 among RGOAs. As described in the BET and SEM analyses, changes in GO or RGO ratios in graphene aerogels resulted in changes in the C/O ratios after pyrolysis as they changed the interaction mechanism between the reactants used during the synthesis.

**Figure 5:**
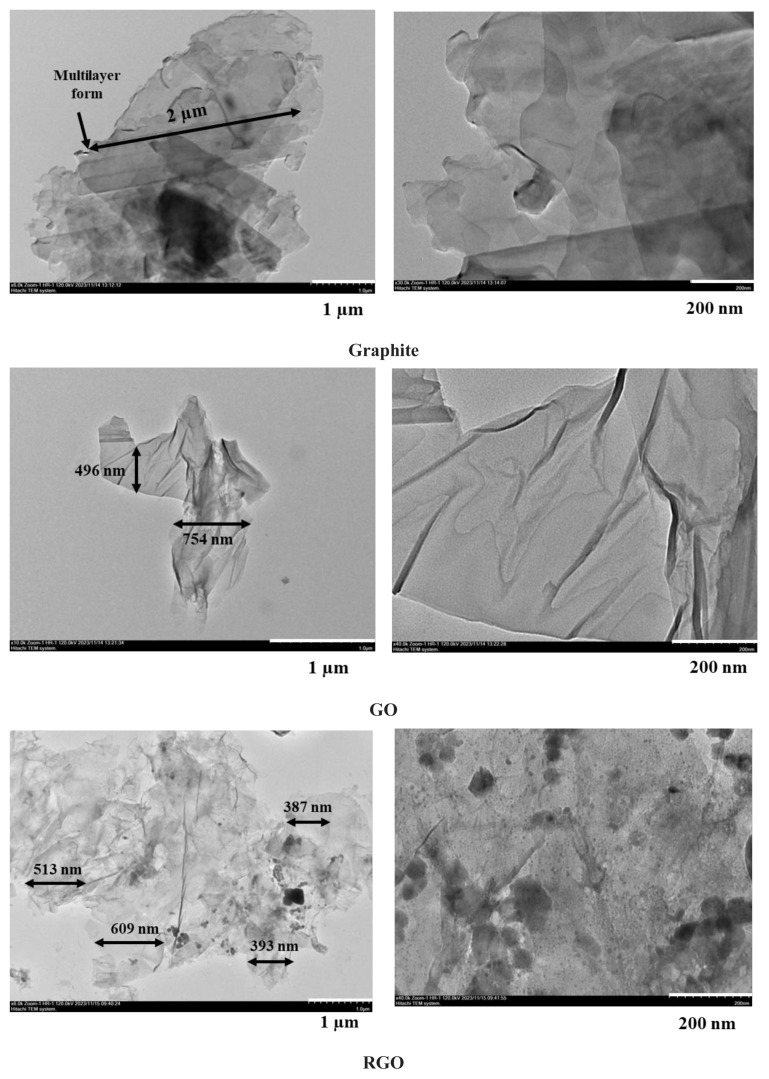

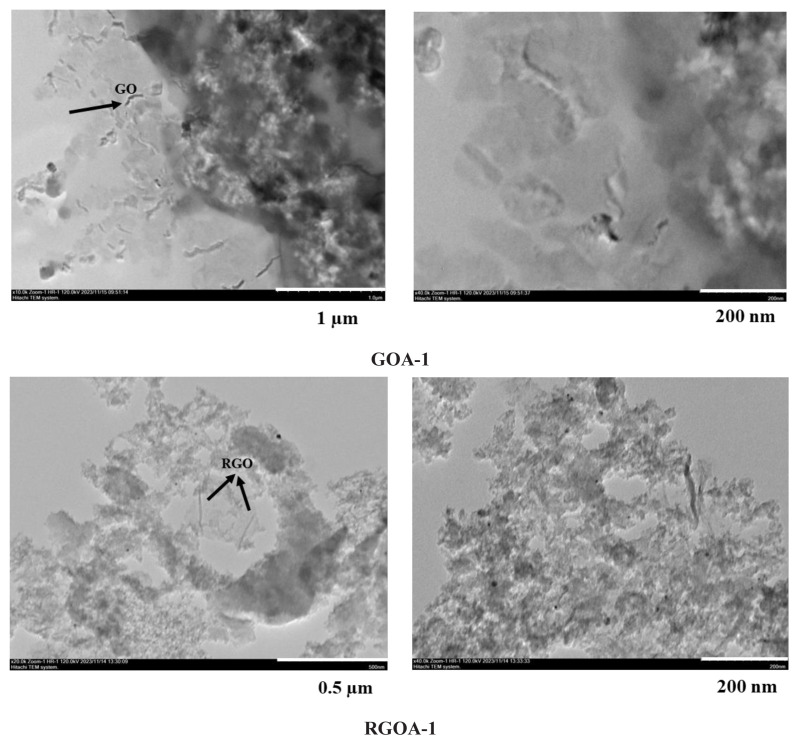
TEM images of commercial graphite, GO, RGO, GOA-1 and RGOA-1.

TEM images of graphite, GO, RGO, GOA-1, and RGOA-1 materials are presented in Figure 5. The size measurements indicated on the images were obtained using the ImageJ program. In TEM images, the multilayer form of graphite and the flaked form of GO are clearly seen. Compared to the crumpled paper form of GO, the structure of thermally reduced graphene oxide is severely expanded and highly distorted, which can damage the structure of the material [30]. The size of RGO sheets has decreased compared to GO sheets. In summary, graphite, GO, and RGO structures are transparent and the lateral dimensions of the layers range from hundreds of nanometers to several micrometers [57]. In addition, in the TEM images of GOA-1 and RGOA-1 aerogels, as shown in the SEM images, carbon particles, generally in spherical form, formed an interconnected network structure with nonuniform textural structure. It is clearly seen that GO or RGO layers are distributed within this aerogel matrix [58].

**Figure 6:**
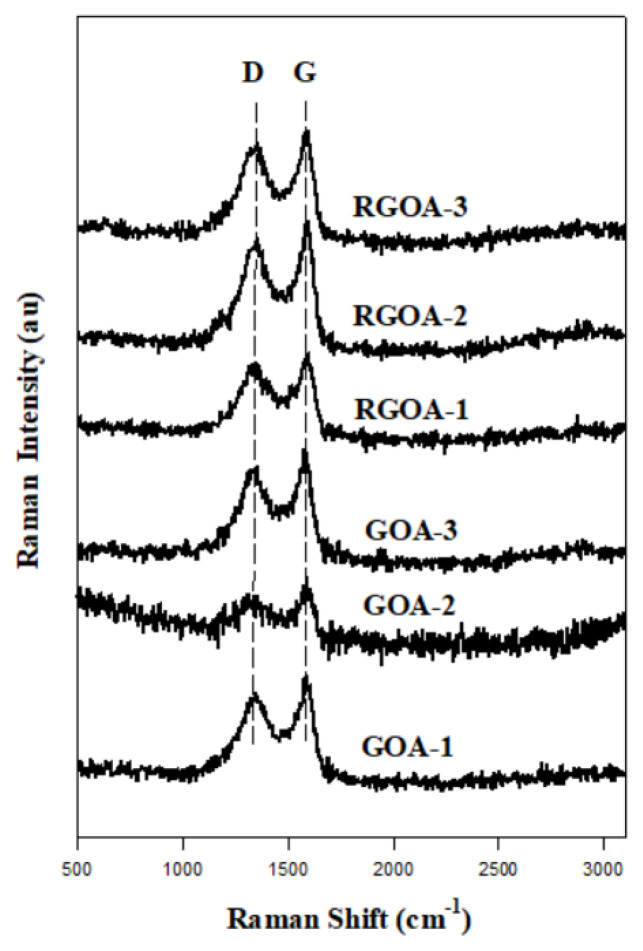
Micro-RAMAN spectra of graphene aerogels.

The RAMAN spectra of graphene aerogels are presented in Figure 6. The regions of the D and G bands on the spectra are in harmony with each other on a linear line. D peaks of graphene aerogels became evident at 1300 cm^−1^, and G peaks at 1500 cm^−1^. The G peak in the spectrum gives information about the sp^2^ (C=C) hybridization by showing the graphitization degree, and the D peak gives information about the irregular carbon structures in the lattice of sp^3^ (C-C) hybridized carbon atoms. Therefore, the peak intensity of the D band is often used as a measure of the degree of disturbance. The value ratio between the D band and the G band (ID/IG) is used to determine the degree of disorder in the lattice structure of graphene [59].

Information on RAMAN spectra are summarized in Table 4. The ID/IG ratio shows the different degrees of graphitization of the samples. The ID/IG ratio of RGO has a value between graphite and GO. The amount of functional groups on graphene sheets is proportional to the ID/IG values. High ID/IG indicates the presence of interaction between graphene sheets and R-F polymers within graphene aerogels. This is explained by the increase in sp^2^ fields [26,50]. This explains that the sp^2^ hybridization areas in RGO-derived graphene aerogels are lower than in GO-derived graphene aerogels. In other words, the graphitization degree of RGOAs is lower than that of GOAs.

**Figure 7:**
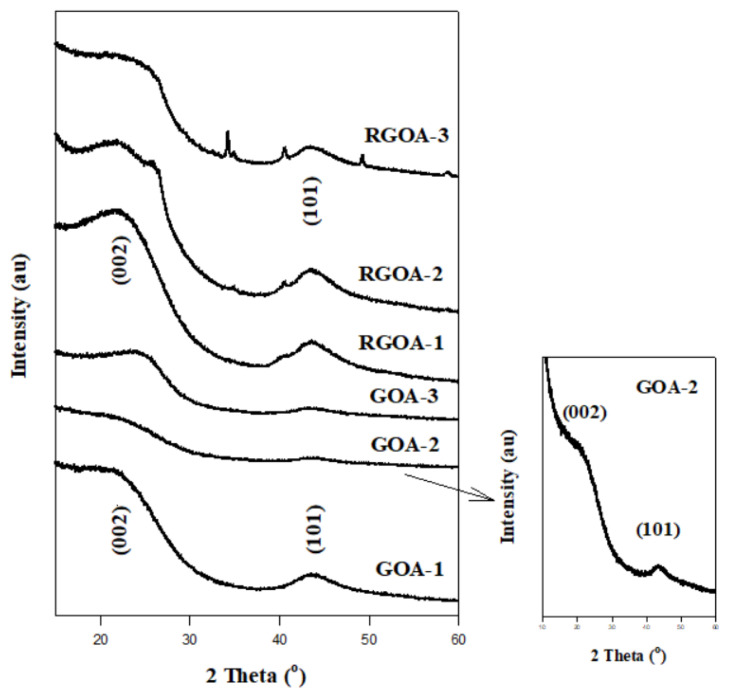
XRD spectra of graphene aerogels.

XRD spectra of graphene aerogels are presented in Figure 7. Graphene aerogels show two diffraction peaks at approximately 2θ = 22.5° and 2θ = 43.5°. These diffraction peaks correspond to the crystal planes (002) and (101), respectively [49]. These two peaks indicate that the graphene aerogels contain partially graphitized carbon particles [26]. These peaks are sharper in RGOAs than in GOAs. This is due to the fact that the RGO sheets in the structure of RGOAs become more pronounced as the amount increases, as shown in the SEM images, unlike GO. In addition, as the amount of RGO in the RGOs increased, weak diffraction peaks appeared at 2θ values of 40.5° 49.3°. These weak peaks correspond to weak graphitic (100) and (101) planes formed after pyrolysis, respectively [60].

The concept of interlayer distances for graphene-based materials is based on Bragg’s law and is expressed as [61]


(1) $$n\lambda =2d_{\left(hkl\right)}\;\text{sin }\theta ,$$

where λ is the wavelength of the X-ray (1.5016 Å), θ is the scattering angle, n is an integer representing the order of the diffraction peaks, d is the interlayer distance of the lattices, and (hkl) is the Miller indices. The thickness of the graphene can be estimated using Sherrer equation [61,62]:


(2) $$D_{hkl}=\frac{K\lambda }{B\;\text{cos}\;\theta },$$

where D_hkl_ is the size of the crystal (L_c_ is the vertical size of crystallites for the (002) peak, L_a_ is the lateral size of the crystallite for the (100) peak), K (0.89 for the (002) peak and 1.84 for the (101) peak) is a constant that depends on the shape of the crystal, and B is the full width at half maximum (FWHM). The number of graphene layers (N_GR_) is obtained using Equation 3 [62].


(3) $$N_{GR}=\frac{D_{002}}{d_{002}}$$

The XRD spectrum data of the Miller indices (002) and (101) of GOA, and RGOA are summarized in Table 5. A linear change in d, D and N values was observed as the GO ratio increased in GOAs. In RGOAs, d values were not affected by the change in the RGO ratio in their content.

The characterization of carbon surfaces is carried out by many techniques. The cyclic voltammetry (CV) technique is widely used in the study of electrochemical reactions on individual electrodes. Most commonly, a three-electrode cell with liquid electrolyte is used. In this study, the CV technique was used to examine the electrochemical behavior of graphene-based materials and graphene aerogels surfaces against corrosion. The synthesized materials were subjected to corrosion at a constant potential of 1.2 V for 24 h. CV measurements were carried out both before and after corrosion.

**Figure 8:**
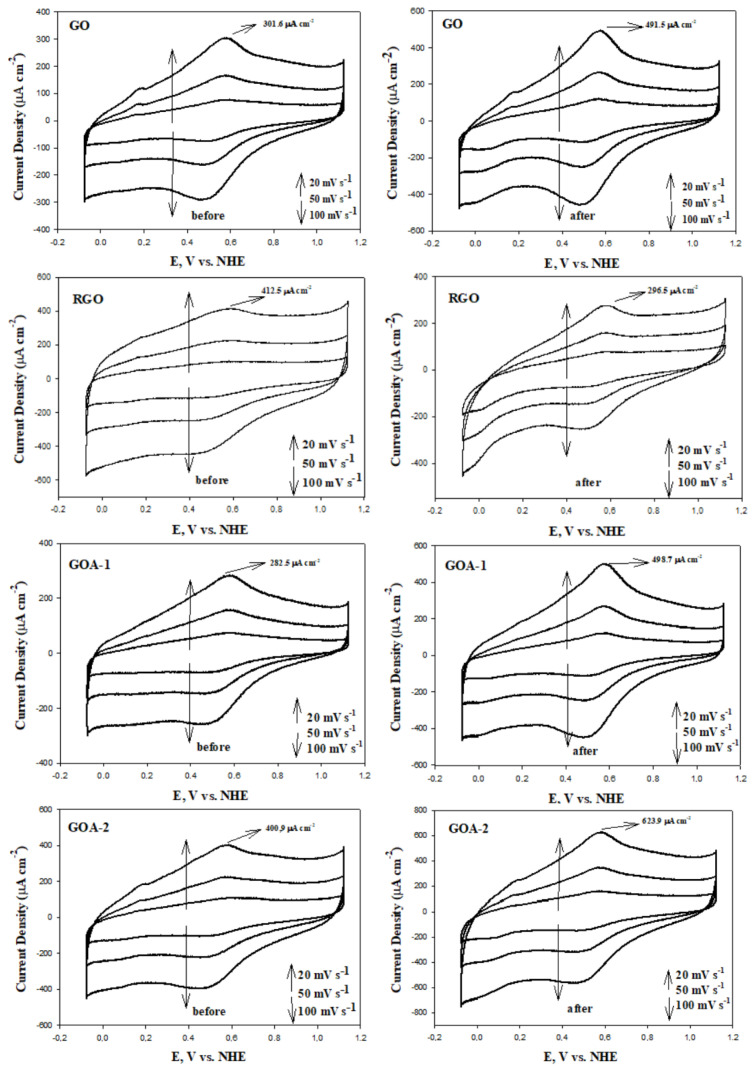

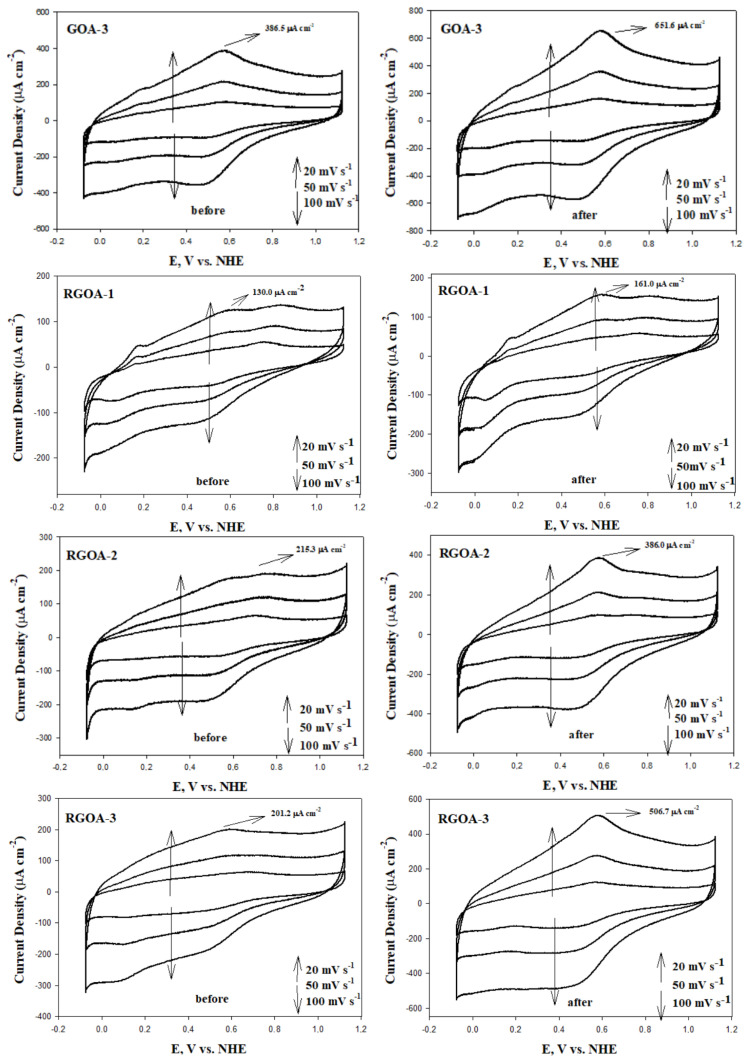
Cyclic voltammograms before and after carbon corrosion of graphene-based materials and graphene aerogels (potential range: −0.28 to 0.92 V; scan rate: 20, 50, 100 mV s^−1^; electrolyte: N_2_ saturated 0.1 M HClO_4_ solution).

The CV voltammograms of the synthesized materials before and after corrosion, obtained at scan rates of 20 mV s^−1^, 50 mV s^−1^, and 100 mV s^−1^, are presented in Figure 8. The upper region of the CV voltammograms represents the anodic current density, while the lower region represents the cathodic current density. The anodic and cathodic current regions are associated with the presence of electroactive groups on the carbon surface [63]. It was observed that the anodic and cathodic current densities increased as the scan rate increased in all the synthesized materials. GOAs have the highest anodic and cathodic current densities before corrosion. This is due to the excess of electroactive sites compared to RGOAs because of the presence of oxygen-containing functional groups on the surface of GOAs. After electrochemical corrosion, the anodic and cathodic current densities of all graphene aerogels increased within the range of 400 to 800 mV. The surface oxides formed due to the increased oxygen content on the carbon surface after corrosion also increased the electroactive regions responsible for the anodic and cathodic peaks. Studies on this subject have been conducted in the literature. Many researchers have examined the electrochemical reactions of carbon oxide groups formed on carbon surfaces after corrosion. The most probable redox processes on carbon surfaces have been identified as particularly quinone and hydroquinone (Q - HQ) redox systems. These redox reactions increased the anodic and cathodic current density [63]. In addition, numerical values of anodic current densities of graphene aerogels are indicated on CV graphs. It is seen that the effect of corrosion on the graphene surface is related to the physical properties of the materials.

**Figure 9:**
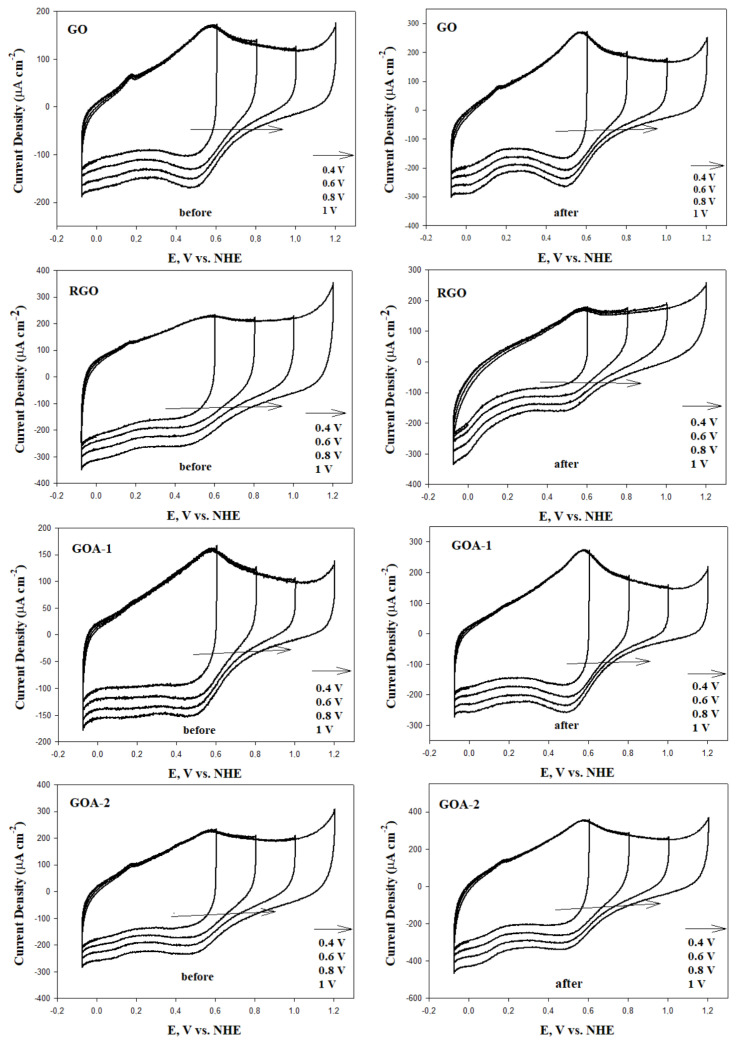

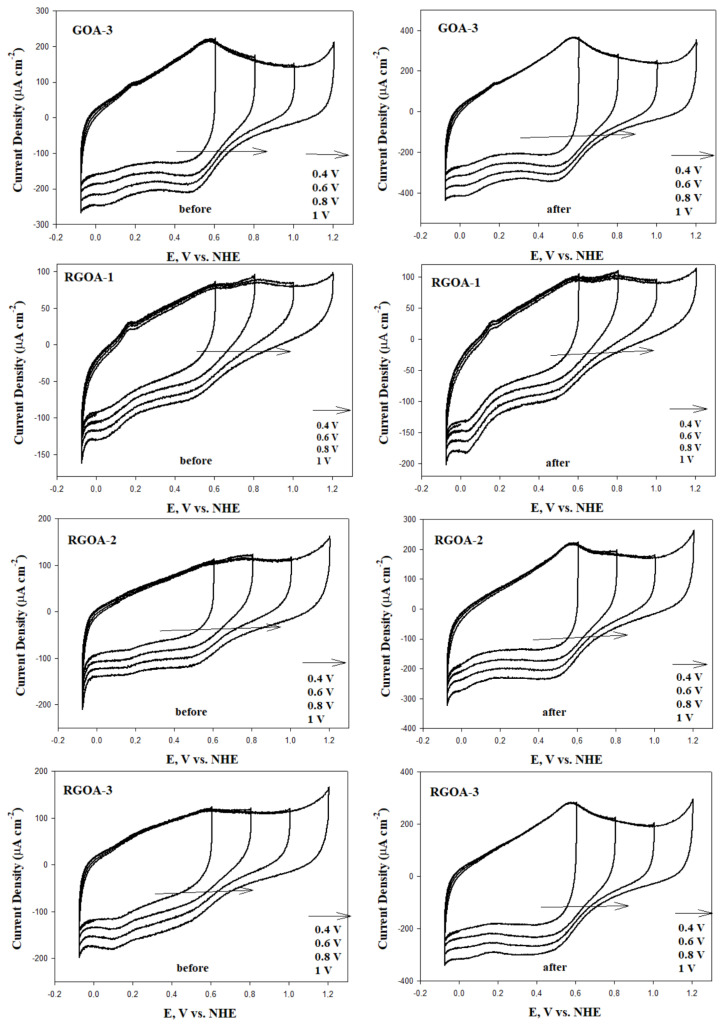
Cyclic voltammograms before and after carbon corrosion of graphene-based materials and graphene aerogels (potential range: −0.28 to 0.4 V, −0.28 to 0.6 V, −0.28 to 0.8 V, −0.28 to 1 V; scan rate: 50 mV s^−1^; electrolyte: N_2_ saturated 0.1 M HClO_4_ solution).

The CV curves obtained at different potential ranges of the synthesized materials are presented in Figure 9. Voltammograms appear to be almost rectangular curves. This is explained by the fact that the redox reactions taking place on the carbon surface have a pseudo-capacitive effect. The higher the potential upper limit, the greater the conductivity of the materials. This simplifies the load transfer mechanism. This also improves the Q-HQ redox systems by increasing the redox reactions on the material surface. In this way, the areas of the anodic and cathodic current regions are increased. These features are particularly ideal for energy storage systems [64,65]. In order to determine the corrosion rate occurring on the surfaces of the samples after corrosion, the amount of Q-HQ redox couples formed on the surface after corrosion was calculated. The charge observed due to the *C* = *O* + *e*^−^ + *H*^+^ ↔ *C* – *OH* reaction represents the so-called capacitance charge formed after corrosion [66]. The capacitances of the synthesized materials before and after corrosion were calculated in the potential range of −0.28 to 0.92 V, at a scan rate of 100 mV s^−1^. The difference in capacitance values between before and after corrosion is explained as the pseudo-capacitive charge on the sample surface. Calculated values are presented in Table 6.

Specific capacitance values were calczulated using Equation 4 [64].


(4) $$C_{S}=\int \frac{\left(IdV\right)}{\left(vm\Delta V\right)}$$

In Equation 4, C_S_ is the specific capacitance, I is the current value, V is the potential window, υ is the scan rate, and m is the active mass used in the working electrode. The amount of charge occurring on the surface was calculated using Equations 5 and 6 [67].


(5) $$C_{S}=\frac{dQ}{dV}=\frac{I}{\left(\frac{dV}{dt}\right)}$$

(6) $$Q(mC\;{cm}^{-2})=\int Idt=\int I/(dV/dt)dV$$

Here, Q is the charge, mC is the electric charge unit (millicoulomb), I is the current density (μAcm^−2^), and dV/dt is the scanning speed (mV s^−1^).

**Figure 10:**
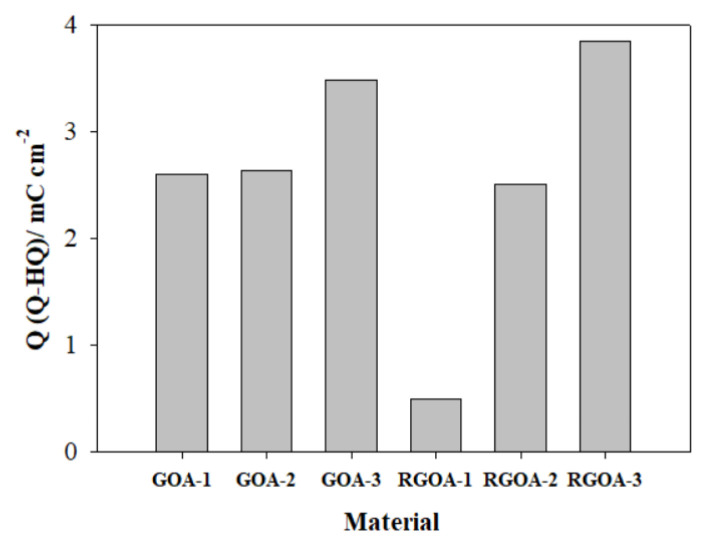
Pseudo-capacitive charge amounts formed on the surface of graphene aerogels after corrosion.

The material with the highest C_S_ value before corrosion is RGO with 227.5 Fg^−1^. Among graphene aerogels, the highest C_S_ value belongs to GOAs. After corrosion, the surface of all materials except RGO was oxidized. As the ratio of GO or RGO in graphene aerogels increased, the rate of being affected by corrosion increased. The amount of pseudo-capacitive charge formed on the surface of graphene aerogels after corrosion is summarized as a bar graph in Figure 10. The lowest pseudo-capacitive charge ratio belongs to RGOA-1 aerogel with 0.5 mC cm^−2^.

Since RGO was morphologically damaged after corrosion, its specific capacitance value decreased and there was no pseudo-capacitive charge formation on its surface. This is due to the fact that RGO is more hydrophobic and chemically inert than GO [29]. Additionally, RGO has larger sheet surfaces than GO. Due to these properties of RGO, the surfaces of metals or alloys are coated with RGO sheets to increase the resistance of the surfaces against corrosion. Bagherzadeh et al. grew GO as electrochemically reduced graphene oxide nanosheets (RGON) on a carbon steel alloy. It is concluded that the presence of RGON coating provides corrosion protection on the carbon steel surface. They attributed the corrosion protection ability of graphene coating on carbon steel to its large specific surface area, excellent mechanical properties, and two-dimensional geometry of the graphene sheet [68].

**Figure 11:**
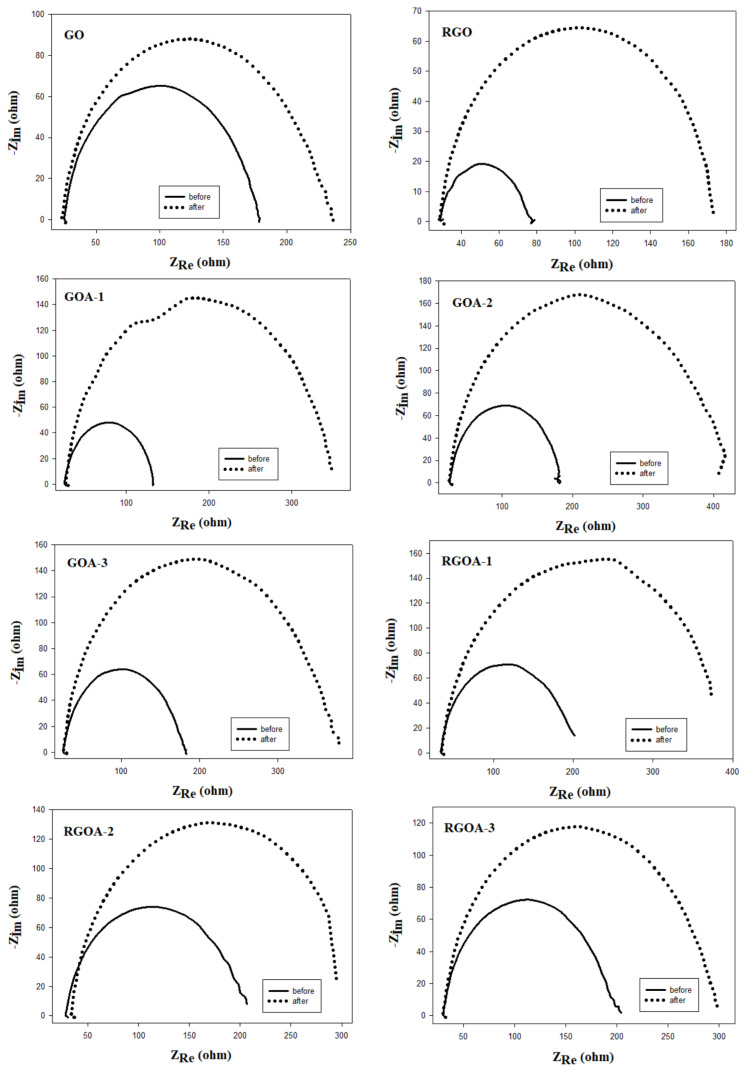
Nyquist plots before and after carbon corrosion (frequency range: 10^5^–1 Hz; potential: 0.9 V; N_2_ saturated 0.1 M HClO_4_ solution) and the electrical equivalent circuit model.

EIS tests were carried out to examine the electrochemical dynamic properties of ion transfer at the solid/liquid interface surface of the synthesized materials. These tests were performed within the frequency range of 10^5^ to 1 Hz. In Figure 11, Nyquist graphs obtained before and after carbon corrosion obtained at 0.9 V are presented. Nyquist plots for all materials exhibited a semicircular shape in the midfrequency region. These Nyquist plots are compatible with Randles cell models, which are one of the electrical equivalent circuit models. The Randles cell is a simple and convenient combination of a capacitor (double-layer capacitor (C_dl_)) and two resistors (electrolyte resistance (R_S_), charge transfer (R_ct_) or polarization resistance (R_P_)) [69]. The semicircles here represent the R_ct_. The smaller the size of the semicircle, the lower the R_ct_, and therefore, the faster the transfer of ions and electrons [24,51]. That is, this semicircle size is related to the kinetics of faradaic reactions at the electrode-electrolyte interface. After corrosion, the R_ct_ of the synthesized materials at the electrode-electrolyte interface increased. In this case, the corrosion process causes the rate of faradaic reactions to decrease.

The results of the Nyquist plots are summarized in Table 7. In these plots, the first point where the high frequency region intersects with the real axis gives the R_S_. It is seen that the R_S_ values are very close to each other, indicating that the electrolyte used in these analyses is the same. The end point of the semicircle is the low frequency region and represents the R_ct_. The diameter of the semicircle gives the R_P_. In other words, the size of the diameter of the semicircle affects both the R_ct_ and the R_P_ [36]. Graphene aerogels with the highest load transfer resistance before corrosion are GOAs. After corrosion, the electrode-electrolyte interface is the most affected by corrosion, again GOA. This indicates that RGOAs are more resistant to corrosion treatment.

**Figure 12:**
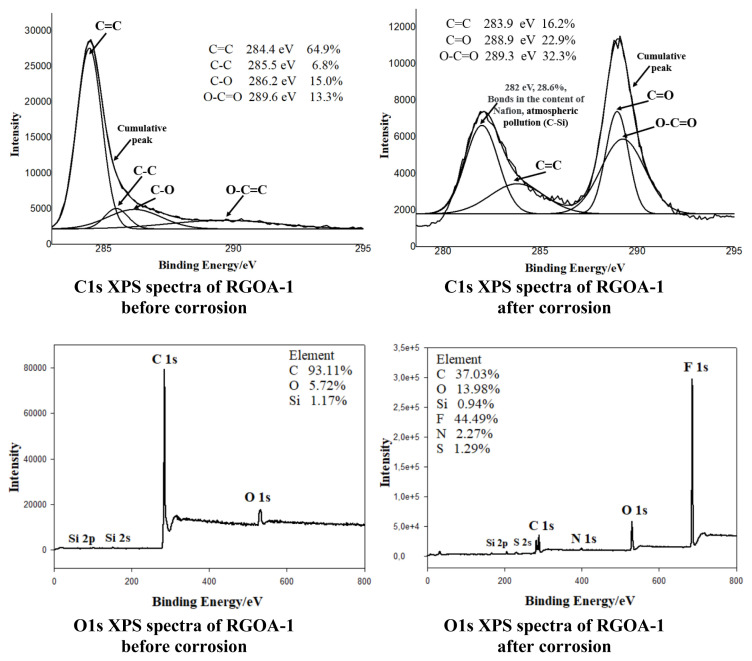
XPS spectra of RGOA-1 before and after corrosion.

To examine the corrosion behavior and chemical composition of the RGOA-1 aerogel surface, XPS analyses before and after corrosion were performed and are presented in Figure 12. There are four peaks in the before-corrosion C1s spectrum of RGOA-1 at 284.4 eV, 285.5 eV, 286.2 eV, and 289.6 eV. These peaks represent C=C, C-C, C-O, and O-C=O bonds, respectively [21]. Additionally, before corrosion, approximately 65% of C=C bonds are present in RGOA-1. There are four peaks in the after-corrosion C1s spectrum of RGOA-1 at 282 eV, 283.9 eV (C=C), 288.9 eV (C=O), and 289.3 eV (O-C=O) [70]. It is observed that the peak intensity of the C=C bond decreases considerably after corrosion. The peak region at 282 eV represents atmospheric pollution and the chemical bonds of Nafion in the electrode solution [71,72]. This is also evident in the O1s XPS spectra of RGOA-1. In the O1s XPS spectrum of RGOA-1 after corrosion, the presence of elements such as fluorine (F), nitrogen (N), and sulfur (S), especially C, in the structure of Nafion is indicated [71]. A very small amount of atmospheric pollution, Silicon (Si), is also present in the O1s XPS spectra of RGOA-1 before and after corrosion [72]. While The C/O (atomic ratio) of RGOA-1 was 16.3% before corrosion, this ratio decreased to 2.65% after corrosion. It is evident that the RGOA-1 aerogel is affected by corrosion.

**Figure 13:**
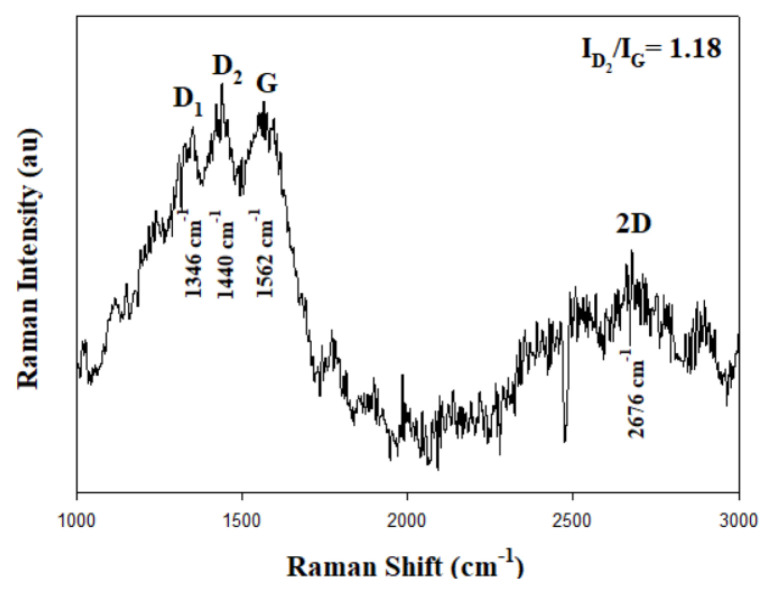
Micro-RAMAN spectra of RGOA-1 after corrosion.

Raman analysis of RGOA-1 aerogel after corrosion is presented in Figure 13. Most studies have attributed the G band (approximately 1562 cm^−1^) to crystalline graphite and the D bands (1346 cm^−1^ and 1440 cm^−1^) to the structural disorder present in the graphite structure. D bands in different regions of the spectrum arise from the multiple structural topographies of disordered carbon materials [73]. While the D bands of RGOA-1 aerogel before corrosion showed only one peak, they showed two peaks after corrosion. In addition, while the 2D peak of RGOA-1 aerogel is not present before corrosion, it is seen as a single peak at 2676 cm^−1^ Raman shift value after corrosion. The 2D peak for a perfectly stacked few-layer graphene (about 6–8 layers) is reported to exhibit a two-peak profile. These two peak profiles represent a 2D overtone and G + D combination, respectively [74]. As the distortion increases, the 2D band forms as one peak. This is due to the advancement of turbo stratification and the disappearance of 3D ordering [73]. As a result, while the ID/IG ratio of RGOA-1 aerogel was 0.86 (Table 4) before corrosion, this value increased to 1.18 after corrosion. This shows that sp^2^ hybridization after corrosion decreases considerably and the aerogel undergoes structural deterioration after corrosion [59].

## 5. Conclusion

Graphene aerogels are ideal 3D electrode materials used in applications where energy is stored and converted. Various methods have been applied in the synthesis of graphene aerogels. In all these methods, GO is used as the graphene-based precursor material. In this study, unlike the literature, both GO-derived and RGO-derived aerogels were synthesized. The synthesis was carried out using the sol-gel method and SCCO_2_ drying. Since GO and RGO contain functional groups containing different amounts of oxygen, the crosslinking mechanisms also occur differently. This situation considerably changes the physical and chemical properties of the synthesized aerogels. SEM images of aerogels demonstrate that each aerogel has a unique morphological structure. The plates were embedded in the aerogel due to the hydrophilic nature of GO in the GOAs. In RGOs, on the other hand, as the number of plates increased due to the more hydrophobic structure of RGO, RGO plates became more pronounced in the structure. This is evident in XRD analysis. In the syntheses, the surface area decreased as the amount of GO in the aerogel increased, while it increased as the amount of RGO increased. When the amount of graphene-based material in the aerogel was 1% by weight, the surface area of GOA-3 was 273.91 m^2^ g^−1^, while the surface area of RGOA-3 was 923.43 m^2^ g^−1^. To examine the electrochemical behavior of the synthesized graphene aerogels against corrosion, the surfaces of the aerogels were exposed to carbon corrosion for 24 h. Among the graphene aerogels, the highest C_S_ value belongs to GOAs. The aerogel most resistant to corrosion was RGOA-1. The pseudo-capacitive charge ratio of RGOA-1 after corrosion was 0.5 mC cm^−2^. XPS and Raman analyses were performed on RGOA-1 aerogel before and after corrosion. As a result, while the C/O (atomic ratio) of RGOA-1 was 16.3% before corrosion, this ratio decreased to 2.65% after corrosion. While ID/IG ratios were 0.86 before corrosion, this value increased to 1.18 after corrosion.

## Figures and Tables

**Table 1:** BET analysis results of materials.

Material	BET surface area (m^2^ g^−1^)	Average pore diameter (nm)	t-plot micropore area (m^2^/g)	t-plot micropore volume (cm^3^ g^−1^)	Average particle size (nm)	BJH pore volume (cm^3^ g^−1^)	D-H pore volume (cm^3^ g^−1^)
**Graphite [36]**	25.25	5.51	-	-	237.64	0.053	0.063
**GO [36]**	10.51	4.85	-	-	570.93	0.016	0.020
**RGO [36]**	213.37	13.28	-	-	28.12	1.287	1.411
**GOA-1**	470.56	4.06	245.01	0.123	12.75	0.345	0.339
**GOA-2**	418.16	3.26	178.03	0.123	14.35	0.229	0.225
**GOA-3**	273.91	2.87	170.82	0.085	21.91	0.137	0.136
**RGOA-1**	668.77	3.61	119.5	0.056	8.97	0.619	0.592
**RGOA-2**	829.64	7.11	162.28	0.077	7.23	1.439	1.430
**RGOA-3**	923.43	6.39	148.49	0.067	6.50	1.585	1.575

**Table 2:** Surface areas of graphene aerogels synthesized by sol-gel method in the literature.

Sample	Reaction solution	Drying method	Pyrolysis conditions	BET surface area, m^2^ g^−1^	Mesopore volume, cm^3^ g^−1^	Micropore volume, cm^3^ g^−1^	Ref.
**RGOA-3**	Sol-gel polymerizationwater, resorcinol, formaldehyde, Na_2_CO_3_ catalyst, **RGO** (wt. 1% RGO)	SCCO_2_	1000 °C, 4 h, N_2_ atmosphere	923.43	-	0.067	Our study
**GOA-1**	Sol-gel polymerization water, resorcinol, formaldehyde, Na_2_CO_3_ catalyst, GO (wt. 0.25% GO)	SCCO_2_	1000 °C, 4 h, N_2_ atmosphere	470.56	-	0.093	Our study
**4 wt.% RF**	Sol-gel polymerization water, resorcinol, formaldehyde, Na_2_CO_3_ catalyst, GO (wt. 4% RF)	SCCO_2_	1050 °C, 3 h, N_2_ atmosphere	584	2.9	-	[48]
**2 wt.% RF**	Sol-gel polymerization water, resorcinol, formaldehyde, Na_2_CO_3_ catalyst, GO (wt. 2% RF)	SCCO_2_	1050 °C, 3 h, N_2_ atmosphere	762	3.3	-	[48]
**C10**	Sol-gel polycondensation, water, resorcinol, formaldehyde, Na_2_CO_3_ catalyst, GO (wt. 10% GO)	SCCO_2_	800 °C, N_2_ atmosphere	812	0.58	0.33	[27]
**CGO/RF-10**	Self-assembly, water, resorcinol, formaldehyde, Na_2_CO_3_ catalyst, GO (wt. RF:GO–10:1)	Freezing	900 °C, 3 h, N_2_ atmosphere	303	0.58	-	[26]
**CGO/RF-25**	Self-assembly, water, resorcinol, formaldehyde, Na_2_CO_3_ catalyst, GO (wt. RF:GO–25:1)	Freezing	900 °C, 3 h, N_2_ atmosphere	680	0.36	-	[26]
**GCPFCX-10**	Sol-gel polymerization water, Phenol, formaldehyde, KOH catalyst, GO (wt. 10% GO)	Under ambient pressure (80 °C)	800 °C, 2 h, N_2_ atmosphere	378	0.56	-	[49]
**GCPFCX**	Sol-gel polycondensation water, phenol (P), formaldehyde, KOH catalyst, GO (wt. GO:P–1:9)	Under ambient pressure (80 °C)	800 °C, 2 h, N_2_ atmosphere	395	0.69	-	[25]
**RF-GOA1**	Sol-gel polymerization water, phenol, formaldehyde, KOH catalyst, amine-functionalized GO (wt. 1% GO)	Under ambient pressure (100 °C)	-	266	0.61	0.02	[50]
**GCCA-300**	Sol-gel polycondensation water, resorcinol, formaldehyde, Na_2_CO_3_ catalyst, GO (wt. RF/GO=300)	Under ambient pressure	1000 °C, 3 h, N_2_ atmosphere	820.9	0.119	0.119	[51]
**GCCA-100**	Sol-gel polycondensation water, resorcinol, formaldehyde, Na_2_CO_3_ catalyst, GO (wt. RF/GO=100)	Under ambient pressure	1000 °C, 3 h, N_2_ atmosphere	475.5	0.034	0.034	[51]
**GO–RF-0.5**	Sol-gel polymerization water, resorcinol, formaldehyde, Na_2_CO_3_ catalyst, GO (wt. 0.5% GO)	Under ambient pressure (40 °C)	-	248	1.44	-	[52]
**GO–RF-1**	Sol-gel polymerization water, resorcinol, formaldehyde, Na_2_CO_3_ catalyst, GO (wt. 1% GO)	Under ambient pressure (40 °C)	-	223	0.79	-	[52]
**CAG-0.75**	In situ sol-gel polymerization and inverse emulsion Water, resorcinol, formaldehyde, GO (wt. 0.75% GO)	Under ambient pressure	700 °C, 2 h, N_2_ atmosphere	488	0.158	0.215	[53]
**CAG-0.15**	In situ sol-gel polymerization and inverse emulsion, water, resorcinol, formaldehyde, GO (wt. 0.15% GO)	Under ambient pressure	700 °C, 2 h, N_2_ atmosphere	468	0.294	0.197	[53]
**CA-A1**	Sol–gel polymerization, water, Phenol, formaldehyde, nitric acid and NaoH solution, GO (wt 2% GO)	Under ambient pressure	1073 °C, 2 h, N_2_ atmosphere	607	1.12	0.12	[54]
**CA-C1**	Sol-gel polymerization, water, Phenol, formaldehyde, nitric acid and NaoH solution, GO (wt. 0.5% GO)	Under ambient pressure	1073 °C, 2 h, N_2_ atmosphere	487	0.03	0.23	[54]

**Table 3:** The C/O ratios of precursor graphene-based materials and graphene aerogels.

Material	C/O	
	Weight	Atomic
**Graphite**	8.56	11.41
**GO**	0.62	0.83
**RGO**	2.04	2.72
**GOA-1**	21.77	29.03
**GOA-2**	4.88	6.50
**GOA-3**	10.22	13.62
**RGOA-1**	10.00	13.31
**RGOA-2**	12.87	17.12
**RGOA-3**	7.03	9.36

**Table 4:** Micro-RAMAN spectra of graphene-based materials and graphene aerogels.

Material	Raman shift (cm^−1^)		D peak intensity (au)	G peak intensity (au)	ID/IG
	D band	G band			
**Graphite [36]**	1333.87	1562.1	847.5	1347.75	0.63
**GO [36]**	1347.35	1606.8	1932	1674	1.15
**RGO [36]**	1371.65	1587.14	424	440.5	0.96
**GOA-1**	1344.9	1584.25	590.95	771.33	0.77
**GOA-2**	1316.6	1591.95	385.2	604.1	0.64
**GOA-3**	1359.28	1570.62	604.07	831.66	0.73
**RGOA-1**	1349.12	1591.95	541.01	625.46	0.86
**RGOA-2**	1350.24	1592.05	764.32	946.67	0.81
**RGOA-3**	1359.28	1581.79	681.91	809.26	0.84

**Table 5:** Data of XRD spectra of GOAs, and RGOAs.

Material	(002)				(101)			
	Peak (2θ)	d (Å)	Crystal size, D (L_c_) (Å)	Number of layers, N	Peak (2θ)	d (Å)	Crystal size, D (L_a_) (Å)	Number of layers, N
**Graphite [36]**	26.70°	3.3	58.50	17.5	-	-	-	-
**GO [36]**	11.40°	7.8	22.60	2.9	-	-	-	-
**RGO [36]**	24.70°	3.6	16.20	4.5	-	-	-	-
**GOA-1**	21.78°	4.1	16.12	4.0	43.79˚	2.07	51.73	25.0
**GOA-2**	21.98°	4.0	22.26	5.5	43.36˚	2.09	63.59	30.4
**GOA-3**	23.89°	3.7	27.13	7.3	43.59˚	2.08	78.54	37.8
**RGOA-1**	22.01°	4.0	17.22	4.3	43.79˚	2.07	61.40	29.7
**RGOA-2**	22.21°	4.0	16.41	4.1	43.79˚	2.07	78.60	37.9
**RGOA-3**	22.01°	4.0	18.42	4.6	4.16˚	2.10	74.78	35.6

**Table 6:** Specific capacitance and pseudo-capacitive charge values of the synthesized materials.​

Material	Before corrosion C_S_ (Fg^−1^)	After corrosion C_S_ (Fg^−1^)	Pseudo-capacitive charge Q (mC cm^−2^)
**GO**	128.1	192.9	2.16
**RGO**	227.5	120.6	−1.07
**GOA-1**	119.6	196.4	2.60
**GOA-2**	191.1	269.9	2.64
**GOA-3**	169.0	274.2	3.49
**RGOA-1**	59.9	74.7	0.50
**RGOA-2**	93.6	170.1	2.51
**RGOA-3**	107.6	222.4	3.85

**Table 7:** The electrolyte resistance (R_s_), the load transfer resistance (R_ct_) and the polarization resistance (R_p_) of the materials before and after carbon corrosion.

Materials	Before corrosion			After corrosion		
	R_s_ (Ω)	R_ct_ (Ω)	R_p_ (Ω)	R_s_ (Ω)	R_ct_ (Ω)	R_p_ (Ω)
**GO**	25.82	178.14	152.32	26.48	236.41	209.93
**RGO**	30.33	78.92	48.59	30.82	173.71	142.89
**GOA-1**	28.60	132.04	103.44	30.15	348.52	318.37
**GOA-2**	26.74	174.25	147.51	28.27	407.42	379.15
**GOA-3**	27.25	182.80	155.55	30.39	377.78	347.39
**RGOA-1**	36.80	201.89	165.09	37.61	373.4	335.79
**RGOA-2**	30.69	206.57	175.88	36.40	295.55	259.15
**RGOA-3**	33.16	204.24	171.08	32.64	301.79	269.15
